# Effects of dietary feed supplementation with heat-treated *Lactobacillus sakei* HS-1 on the health status, blood parameters, and fecal microbes of Japanese Black calves before weaning

**DOI:** 10.14202/vetworld.2023.2293-2302

**Published:** 2023-11-19

**Authors:** Naoya Sasazaki, Katsuki Toda, Hiroshi Hasunuma, Daisaku Matsumoto, Urara Shinya, Osamu Yamato, Takeshi Obi, Takashi Higaki, Oky Setyo Widodo, Kunihiko Ishii, Naoki Igari, Daiji Kazami, Masayasu Taniguchi, Mitsuhiro Takagi

**Affiliations:** 1 Department of Veterinary Medicine, Joint Graduate School of Veterinary Medicine, Yamaguchi University, Yamaguchi, Japan; 2 Shepherd Central Livestock Clinic, Akune, Japan; 3 Kagoshima Agriculture Mutual Aid Association, Soo, Japan; 4 Department of Veterinary Medicine, Joint Faculty of Veterinary Medicine, Kagoshima University, Kagoshima, Japan; 5 Department of Veterinary Science, Division of Animal Husbandry, Faculty of Veterinary Medicine, Airlangga University, Surabaya, Indonesia; 6 JADD Co., Ltd. Yokohama, Japan; 7 Daiwa Pharmaceutical Co., Ltd. Tokyo, Japan; 8 Kazami Food Science, Tochigi, Japan; 9 Department of Veterinary Medicine, Joint Faculty of Veterinary Medicine, Yamaguchi University, Yamaguchi, Japan

**Keywords:** anti-Müllerian hormone, feed supplementation, heat-killed lactic acid bacteria, probiotic, serum amyloid A

## Abstract

**Background and Aim:**

Our previous research suggested that heat-killed *Lactobacillus sakei* HS-1 (HK-LS HS-1) is potentially beneficial for improving intestinal microbes and reducing the number of medical treatments. This study aimed to investigate the effect of HK-LS HS-1 as a supplement in milk replacers (MRs) on clinical health during the 1-month preweaning period.

**Materials and Methods:**

Eighteen female calves were randomly assigned to either a group receiving the HK-LS HS-1 supplement (n = 9) or a control group without it (n = 9). We then investigated the effect of including supplementary HK-LS HS-1; 0.2% in MRs twice daily at 09:00 and 16:00 on the health, serum biochemical parameters (measured using an automated biochemical analyzer), and fecal bacteriological changes of preweaning Japanese Black calves at the day of the start of supplementation (before HK-LS HS-1 supplementation; day 0), at weaning (day 30), and at 2 weeks (day 45) and 4 weeks (day 60) after weaning.

**Results:**

During the supplementation period (0–30 days), (1) an increase (p = 0.023) was observed in albumin, and there was a tendency of increase in total cholesterol level in the HK-LS HS-1 group but not in the control group; (2) substantial differences were obtained after the weaning period (30–60 days), although no differences were observed from 0–30 days in both groups. The anti-Müllerian hormone (AMH) level was substantially increased after weaning in the control group. No differences were observed in the amounts of *Coliform* spp. and *Staphylococcaceae* spp. between the two groups; thus, HK-LS HS-1 supplementation had similar antibacterial effects. A significant reduction was observed in the time to weaning of the HK-LS HS-1 group in the field trial.

**Conclusion:**

Supplementation with HK-LS HS-1 from an early stage after birth to weaning is a cost-effective treatment to improve the growth rate of preweaning calves. However, supplementation during only preweaning periods appears to have no beneficial effects on preventing weaning stress, especially in terms of AMH levels.

## Introduction

Prevention of infectious diseases, particularly diarrhea and respiratory diseases, is a major concern in calf management because calves, particularly Japanese Black (JB) calves, do not have sufficiently developed innate and adaptive immune systems in the preweaning period [1, 2]. These immune systems develop gradually during weaning [3]. Therefore, maintaining calf health during the suckling period is vital for ensuring the survival of the calves after weaning. Although progress has been made in developing vaccines and improving herd management practices and treatment protocols, diseases in calves during the preweaning period, such as diarrhea and pneumonia, cause considerable economic losses.

Antibiotics are used in animal farming to prevent such issues, but their use may contribute to human pathogen resistance. Thus, alternatives such as probiotics and prebiotics have been proposed [4–9]. The internationally accepted definition of a probiotic is a live microorganism that confers a health benefit to the host when administered in adequate amounts. Several lactic acid bacteria (LAB) strains belonging to the genera *Lactobacillus*, *Bifidobacterium*, and *Enterococcus* are considered beneficial to the host and have been widely used as probiotics in cattle production [9]. A recent study of newborn calves indicated intestinal colonization by *Lactobacillus* spp. occur during the first 7 days of life [10]. This finding verifies the hypothesis that early LAB colonization of the intestinal ecosystem may decrease pathogen adherence to the intestinal mucosa. Previous studies have indicated that a stable microbial load of *Lactobacillus* spp. has been shown to improve calves’ weight gain and immune competence [9, 11]. However, heat-killed LABs, such *as Lactobacillus acidophilus* or *Lactobacillus plantarum*, not only have a longer shelf life and are easier to store and transport [12] but also increase immunomodulation and stimulate phagocytic activity in macrophages more effectively than viable LABs [13–15]. In addition, we recently reported the effects of dietary supplementation with heat-killed *Lactobacillus sakei* HS-1 (HK-LS HS-1) at 7 days of age, which is potentially beneficial for improving intestinal microbes and reducing the number of medical treatments by day 21 [16]. Weaning is an important step in calf management and is performed using various methods on beef and dairy farms [17, 18]. During weaning, calves are exposed to several stressful factors that can increase disease susceptibility and reduce production, thereby decreasing body weight [17, 19, 20]. Anti-Müllerian hormone (AMH) is secreted by the antral follicles of the ovary and may be positively associated with fertility and reproductive performance of animals after sexual maturation [21]. It reportedly decreases in JB calves with a large standard deviation (SD) at approximately 3 months of age [22]. We assumed that one cause of this typical phenomenon in female JB calves might be weaning stress, such as increased disease susceptibility, at approximately 2–3 months of age. Therefore, if we obtain similar beneficial effects with HK-LS HS-1 supplementation during the preweaning period as we previously reported by Sasazaki *et al*. [16], this may indicate that it is a useful supplement for calves, not only to prevent diseases but also to regulate the AMH levels of each calf during the pre- and post-weaning periods.

This study aimed to investigate the effect of HK-LS HS-1 as a supplement in milk replacers (MRs) on clinical health (frequency of diarrhea or fever) during the 1-month preweaning period. Furthermore, we recorded hematological measurements to monitor the hepatic, renal, and nutritional status of calves, as well as their mineral intake and inflammation status. Furthermore, we analyzed fecal bacteriological changes in calves during the pre- and post-weaning periods (Experiment 1). Finally, we conducted a field trial to examine whether HK-LS HS-1 supplementation could affect the period from birth to weaning in a rearing environment where a nursing robot was introduced (Experiment 2).

## Materials and Methods

### Ethical approval and Informed consent

The study was conducted according to regulations concerning the protection of experimental animals and the guidelines of Yamaguchi University, Japan (No. 40, 1995; approval date: March 27, 2017). The farmers granted verbal agreement after being educated about the study’s purpose and procedures.

### Study period and location

The initial study was conducted from February to July 2021 on 18 female Japanese black calves at a farm located in Kagoshima Prefecture, Japan, and the second experiment was conducted from November 2021 to August 2022 on 228 F1 calves (male and female) in Tochigi Prefecture, Japan.

### First experiment with JB calves (Experiment 1)

From February to July 2021, we studied 18 female JB calves born on a private farm in Kagoshima Prefecture, Japan, where we conducted our previous study [16]. The details of calving and post-colostrum ingestion are described in detail in our previous study [16]. In brief, calves were fed fresh colostrum from their dams within 2 h of birth. After the first feeding, the calves were orally administered a colostrum replacer containing 60 g of immunoglobulin G (Headstart; Bayer Co. Ltd., Tokyo, Japan) mixed with 1 L of warm water and fed by bottle within 6 h of calving. Calves were separated from dams using a calf hutch for 2−12 days (mean: 6.7 ± 3.6 days) after calving for MR feeding based on the calf’s health condition and willingness to feed. The initial volume of MR provided was 3 L (600 g MR)/day, but this was gradually increased to a maximum of 7 L (1,400 g MR)/day by weaning time, regardless of the body weight and sex of the calves. Fresh water and calf starter (total digestible nutrients >76.0%, crude protein >23.0%; Banana Calf, Nippon Agricultural Industry Co., Ltd., Yokohama, Japan) supplemented with minerals and vitamins were provided *ad libitum* during the experimental period.

The calves were randomly assigned to the HK-LS HS-1-supplemented MR (n = 9) or the control MR group (n = 9). HK-LS HS-1 (*Lactobacillus*-KDP^®^; Daiwa Pharmaceutical Co., Ltd., Tokyo, Japan) was administered orally (0.2% HK-LS HS-1, based on a preliminary trial [16]) twice daily at 09:00 and 16:00 from approximately 1 month before the expected weaning day to weaning time at 2 months of age for each calf.

General health, including appetite and fecal consistency, was monitored daily during the experimental period by experienced farm staff, as detailed in our previous study [16]. In addition, veterinarians visited the farm once a week to observe the health of the calves and to check the progress of the experiment. Briefly, enteritis, bronchitis, and pneumonia were diagnosed based on clinical criteria, such as diarrhea (gruel-like or watery feces), fever (rectal temperature >39.5°C), and signs of respiratory disease (severely increased respiratory sounds accompanied by fever and coughing or a grayish-to-yellowish nasal discharge) [6, 16, 23]. The farm staff observed the stool properties of the calves at the AM and PM feedings, and in cases of mild diarrhea with a good appetite, an oral antidiarrheal, which included berberine tannate, phenyl salicylate, acacia yak powder, and torula yeast (Geritomin; Kyoritsu Seiyaku Corp., Tokyo, Japan), was administered after the feeding. When calves showed severe diarrhea or fever with no appetite at feeding time, antibiotics, including penicillin, kanamycin, or oxytetracycline, were injected by the farm staff under the direction of a veterinarian. This study discontinued medical treatment after the calves showed no more signs of diarrhea and maintained a normal body temperature. All treatment data were recorded for each calf.

Blood samples from the jugular vein (10 mL) were collected on the day of the start of supplementation (before HK-LS HS-1 supplementation; day 0), at weaning (day 30), and at 2 weeks (day 45) and 4 weeks (day 60) after weaning to determine the following: complete blood count (assessed on an F-820 system; Sysmex, Kobe, Japan) and blood urea nitrogen (BUN), serum aspartate aminotransferase (AST), γ-glutamyl transferase (GGT), total protein (TP), total cholesterol (T-Cho), glucose (Glu), free fatty acid (FFA), albumin (Alb), calcium (Ca), magnesium (Mg), and inorganic phosphorus (iP) (measured on a Labospect 7080 autoanalyzer; Hitachi, Tokyo, Japan). Serum vitamin A (VA) and vitamin E (VE) levels were measured using a high-performance liquid chromatography system (Shimadzu, Kyoto, Japan) to evaluate changes in the depletion of both vitamins during the experimental period. Serum amyloid A (SAA) concentration was also measured using an automated biochemical analyzer (Pentra C200; HORIBA ABX SAS, Montpellier, France) with an SAA reagent specific for animal serum or plasma (VET-SAA “Eiken” reagent; Eiken Chemical Co., Ltd., Tokyo, Japan). The SAA concentration was calculated using a standard curve generated using a calibrator (VET-SAA calibrator set; Eiken Chemical Co., Ltd.). AMH concentration was measured using a bovine AMH ELISA kit (AnshLabs, Webster, TX, USA), as previously described by Fushimi *et al*. [24]. All serum samples collected from the same calf were analyzed for AMH concentrations using the same assay to prevent inter-assay differences. The tests were performed to monitor hepatic (AST and GGT) and renal functions (BUN), nutritional status (TP, T-Cho, Glu, FFA, and Alb), mineral intake (Ca, Mg, and iP), inflammation (SAA and A/G ratio), and expected antral follicle number in the ovaries (AMH) of the calves in the two groups.

**Figure-1:**
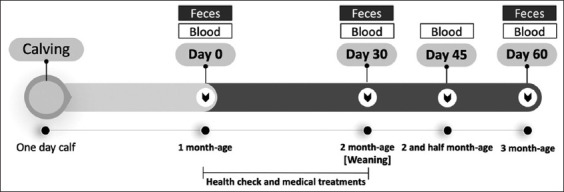
Experimental protocol for calf sampling before and after the weaning period.

A bacteriological analysis was conducted to evaluate the effect of HK-LS HS-1 supplementation. According to a previous method by Yoshida *et al*. [25], fecal microbes were monitored for the number of aerobic bacteria (Coliform group, *Staphylococcaceae*, and *Bacteroides*) in the fecal sample. Fecal samples were collected from all calves on rectal stimulation on days 0 (start of supplementation), 30 (weaning time), and 60 (1 month after supplementation was stopped). The fecal samples (3 g) were immediately placed on ice in a 50 mL conical tube containing 27 mL of brain heart infusion broth medium (Difco; Tokyo, Japan), stirred, and then transported to the laboratory, refrigerated, and processed within 24 h of sampling. The homogenized fecal samples were diluted in modified phosphate-buffered saline with 0.5 g of L-cysteine×HCl×H_2_O, 0.5 g of sodium thioglycolic acid, and 1 g of agar. The relevant dilutions were plated on deoxycholate hydrogen sulfide lactose agar media (Nissui Seiyaku, Tokyo, Japan). The plates were incubated under aerobic conditions at 37°C for 48 h to detect the coliform group. The plates were incubated under anaerobic conditions at 37°C for 48 h for *Bacteroides*. Subsequently, the agar plates were assessed for growth, and the colonies were counted. The total cell counts of *Enterobacteriaceae* per gram of fecal sample were calculated using the relevant calculations for the spiral plater and transformed into log_10_ values. A summary of the experimental protocol for supplementation and sampling is shown in Figure-1.

### Field trial of HK-LS HS-1 supplementation in F1 calves (Experiment 2)

The field supplementation trial of HK-LS HS-1 was conducted with 228 F1 calves born in October (n = 96; male: 57, female: 39), November (n = 72; male: 41, female: 31), and December (n = 60; male: 41, female: 19) of 2021 in Tochigi Prefecture, Japan. After approximately 5 weeks of calf hutch feeding, the calves were moved to group feeding using a milking robot system. The calves were randomly assigned to the HK-LS HS-1-supplemented MR (n = 98, male: 63, female: 35) or control MR groups (n = 130, male: 76, female: 54). For the supplemented calves, two of the five suckling robots operating on the farm were continuously supplied with HK-LS HS-1 (2 g/day) in the MR for 7 months during the test period, and the remaining three suckling robots were not supplied with HK-LS HS-1 supplementation. The managing staff of the herd determined the weaning day according to the continuation of the defined average daily feed intake of the starter for a certain period for each calf. The interval in days between calving and weaning was compared between the two groups.

### Statistical analysis

The results obtained for each group are expressed as the mean ± SD, or standard error of the mean (SEM). Analysis of the values for blood parameters and bacterial count (colony-forming unit, CFU) was conducted using two-way repeated measures Analysis of Variance (with group [supplement or control] and time [0, 30, 45, or 60 d]) followed by a *post hoc* comparison test applying the Bonferroni correction. The days of milk remaining and days of medical treatment between both groups were compared on each sampling day (days 0 and 30, days 30 and 45, and days 0 and 60) using the paired sample Student’s t-test or Mann–Whitney U-test to determine the effects of HK-LS HS-1 on the calves. Results with p < 0.05 were considered statistically significant, while results with p = 0.05–0.1 were considered to indicate a significant tendency.

## Results

### Analysis of the effects of HK-LS HS-1 on each parameter in preweaning calves (Experiment 1)

One calf in the HK-LS HS-1 supplemental group was excluded from the data management because it had an SAA concentration of >50 mg/L (indicating > mean + 2 SD) on day 0, which strongly suggested inflammation at the beginning of the study. Finally, data from eight and nine female calves in the HK-LS HS-1 and control groups, respectively, were included in the study.

### Blood analysis

**Figure-2:**
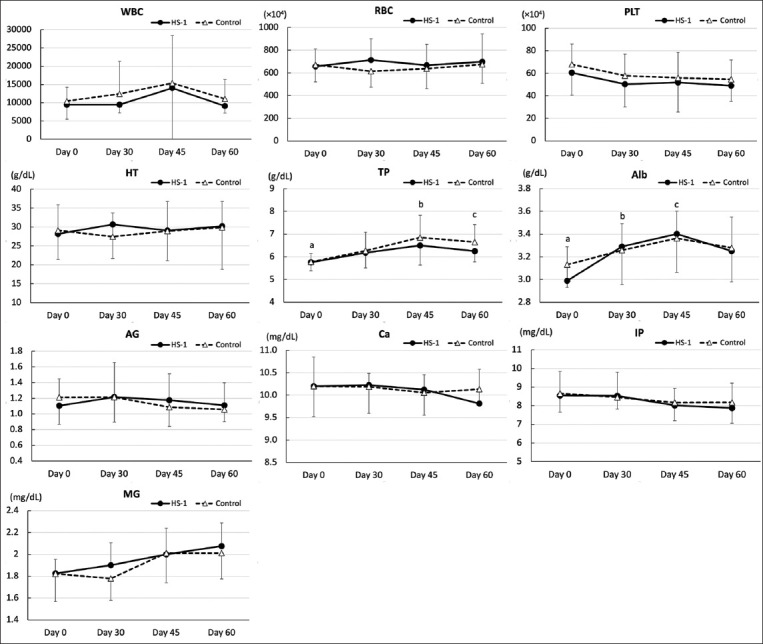
Effects of heat-killed *Lactobacillus sakei* HS-1 (HK-LS HS-1) supplementation on periodic changes in hematology, total protein, albumin, A/G ratio, and ion concentrations (mean ± standard deviation) of calves. TP: ^a-b^Difference between days 0 and 45 in the control group (p = 0.003), and ^a-c^Difference between days 0 and 60 in the control group (p = 0.037). In Alb: ^a-b^Difference between days 0 and 30 in the HK-LS HS-1 group (p = 0.023), and ^a-c^Difference between days 0 and 45 in the HK-LS HS-1 group (p < 0.001).

**Figure-3:**
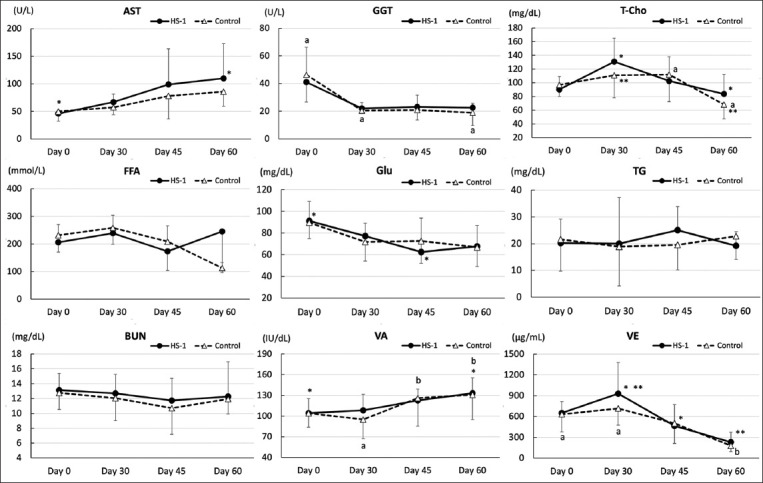
Periodic alterations in serum biochemical parameters (mean ± standard deviation) and vitamins of calves with or without heat-killed *Lactobacillus sakei* HS-1 (HK-LS HS-1) supplementation. FFA is expressed as mean ± SEM. In AST: ^a-b^Difference between days 0 and 60 in the HK-LS HS-1 group (p = 0.032). In GGT: ^a-b^Differences between day 0 and days 30, 45, and 60 in both groups (HK-LS HS-1: p < 0.05, control: p < 0.001). In T-Cho: *Tendency of difference between days 0 and 30 in the HK-LS HS-1 group (p = 0.085). ^a-b^Difference between days 45 and 60 in the control group (p = 0.024). ^a-c^ Difference between days 0 and 60 in both groups (p < 0.05). In Glu: ^a-b^Differences between days 0 and 45 in the HK-LS HS-1 group (p = 0.046). In VA: ^a-b^ differences between days 0 and 60 in the HK-LS HS-1 group (p = 0.041). ^c-d,c-e^Differences between days 30 and 45, and days 45 and 60 in the control group (c-d: p = 0.01, c-e: p = 0.002). * Tendency of difference between days 0 and 60 in the control group (p = 0.052). VE: ^a-b^Differences between days 0 and 60 in the control group (p = 0.024). ^c-d^Differences between days 30 and 60 in the HK-LS HS-1 (p < 0.001) and control groups (p = 0.003). ^c-e^Differences between days 30 and 45 in the HK-LS HS-1 (p = 0.034). *Tendency of difference between days 0 and 60 in the HK-LS HS-1 group (p = 0.068). FFA=Free fatty acid, AST=Aspartate aminotransferase, GGT=γ-glutamyl transferase, T-Cho=Total cholesterol, Glu=Glucose, VA=Vitamin A, VE=Vitamin E.

The results of the hematological and serum biochemical analyses are shown in Figures-2 and 3. No significant differences were observed between the two groups on day 0, indicating that the control and supplemental groups started with similar conditions. No significant differences in all hematological and serum biochemical analyses were observed at any sampling point between the HK-LS HS-1 and control groups. No significant differences were observed between each sampling time within the group with regard to white blood cells, red blood cells, platelets, hematocrit, albumin/globulin ratio, Ca, iP, Mg, FFA, triglycerides, or BUN.

No significant differences in TP levels were observed between days 0 and 30 in either group. In the control group, an increase was observed between day 0 (5.8 g/dL ± 0.4) and both days 45 (6.8 ± 1.0 g/dL: p = 0.003) and 60 (6.6 ± 0.8 g/dL: p = 0.037). Alb levels increased between day 0 (3.0 ± 0.3 g/dL) and both days 30 (3.3 ± 0.2 g/dL: p = 0.023) and 45 (3.4 ± 0.2 g/dL: p < 0.001) in the HK-LS HS-1 supplement group, but not in the control group.

AST was increased in the HK-LS HS-1 supplement group between day 0 (45.9 ± 5.9 U/L) and day 60 (109.7 ± 63.2 U/L: p = 0.032). In contrast, GGT levels on day 30 (22.0 ± 2.7 and 20.4 ± 5.9 U/L), day 45 (23.1 ± 9.5 and 20.8 ± 10.7 U/L), and day 60 (22.5 ± 12.9 and 18.9 ± 6.8 U/L) in both the supplement and control groups, respectively, were decreased compared with those on day 0 (41.0 ± 14.3 and 46.3 ± 20.0 U/L) (supplement; p < 0.05, control; p < 0.01).

T-Cho in the supplement group tended to increase between days 0 (90.1 ± 19.0 mg/dL) and 30 (130.8 ± 34.3 mg/dL: p = 0.085), but not in the control group. A decrease was observed in the supplement group between days 30 and 60 (83.6 ± 29.1 mg/dL: p = 0.020) and in the control group between day 30 (111.0 ± 33.1 mg/dL) and day 60 (68.2 ± 21.1 mg/dL: p = 0.030) and between day 45 (112.0 ± 39.6 mg/dL) and day 60 (p = 0.024). A decrease was observed in Glu levels between day 0 (91.2 ± 18.2 mg/dL) and day 45 (62.4 ± 31.5 mg/dL: p = 0.046) in the supplement group. Increases or tendencies to increase were observed in VA levels between days 0 and 60 in the supplement (104.3 ± 20.9 IU/dL and 133.3 ± 22.0 IU/dL: p = 0.041) and control groups (104.1 ± 20.2 IU/dL and 130.8 ± 35.9 IU/dL: p = 0.052), as well as between days 30 (95.0 ± 28.0 IU/dL) and 45 (126.2 ± 40.8 IU/dL: p = 0.010) and days 30 and 60 (p = 0.002) in the control group. Decreases or tendencies to decrease were observed in VE levels between days 0 and 60 in the supplement (649.6 ± 165.0 and 230.1 ± 143.3 mg/mL: p = 0.089) and control groups (632.4 ± 253.1 and 179.1 ± 85.7 mg/mL: p = 0.024). In addition, a significant difference was observed between day 30 (929.6 ± 446.9 mg/mL) and day 45 (465.6 ± 305.7 mg/mL) (p = 0.034), and between days 30 and 60 (p = 0.003) in the supplement group.

**Figure-4:**
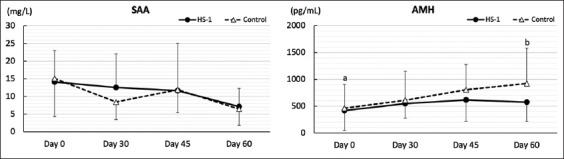
Periodic alterations in serum amyloid A (SAA) and anti-Müllerian hormone (AMH) in heat-killed *Lactobacillus sakei* HS-1-supplemented and control groups. In AMH: ^a-b^Difference between days 0 and 60 in the control group (p = 0.005).

Figure-4 shows the SAA and AMH concentrations in both groups. No significant differences were observed in SAA levels in either group during the sampling days. Although no differences were observed in the AMH level in the supplement group, an increase was observed in the control group between day 0 (462.6 ± 441.9 pg/mL) and day 60 (922.0 ± 658.3 pg/mL: p = 0.005).

### Fecal coliform counts in fecal samples

**Figure-5:**
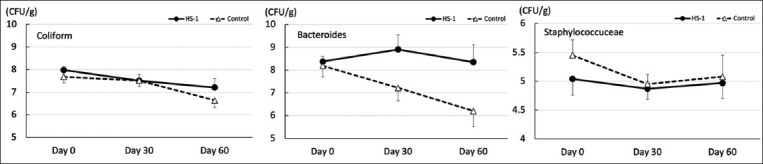
Population of fecal aerobic bacterial (*Coliform* and *Staphylococcaceae*) and anaerobic bacterial (*Bacteroides*) colonies (mean ± standard error of mean) in the heat-killed *Lactobacillus sakei* HS-1 and control groups.

The bacterial counts (CFU/g) in feces from the two groups on days 0, 30 (weaning), and 60 (30 days after weaning) are shown in Figure-5. No significant differences in CFU were observed between the HK-LS HS-1 and control groups at any sampling point. In addition, no differences were observed between the sampling days in the HK-LS HS-1 or control groups.

### Health and medical treatments

Table-1 shows the incidence of illness (diarrhea or fever) and the number of treatments administered during the supplement period from days 0 to 30. Although all nine calves in the control group required medical treatment, three calves in the HK-LS HS-1 supplement group did not receive any medical treatment, and the other five calves received treatments during the supplemental period. The number of calves requiring medical treatment for diarrhea with oral antidiarrheals or antibiotics during the experimental periods was similar between the groups. The mean (± SEM) duration of medical treatment per calf was also similar between the two groups. In contrast, the number of calves left on milk (p = 0.071) and the total number of calves left on milk and having no appetite (p = 0.093) during the supplement period tended to be lower in the supplement group. In addition, the costs of drugs in the HK-LS HS-1 groups (including the cost of HK-LS HS-1; 2.8 g/day × 9 yen/g × 8 calves × 30 days) in total and per calf were 20,834 yen and 2604 yen, respectively, while those of the control group were 26,281 yen and 2920 yen, respectively.

**Table-1:** Efficacy of HK-LS HS-1 supplementation on the number of times appetite decreased, requiring medical treatment, and their frequencies of medical administration.

Variables	HK-LS HS-1 (n = 8)	Control (n = 9)
No. of times appetite decreased (Mean ± SE)	2 (0.25 ± 0.16/calf)*	16 (1.77 ± 0.85/calf)*
No. of times with no appetite (Mean ± SE)	11 (1.38 ± 0.84/calf)	31 (3.44 ± 1.86/calf)
Total (Mean ± SE)	13 (1.63 ± 0.82/calf)**	47 (5.22 ± 2.28/calf)**
No. of oral antidiarrheals time (Mean ± SE)	49 (6.13 ± 2.63/calf)	59 (6.56 ± 2.61/calf)
No. of antibiotics injections times (Mean ± SE)	28 (3.50 ± 1.87/calf)	47 (5.22 ± 1.58/calf)
Total (Mean ± SE)	77 (9.63 ± 4.00/calf)	106 (11.78 ± 2.45/calf)
No. of calves with treatments	5	9
Total no. of treatment days (Mean ± SE)	45 (5.63 ± 2.30/calf)	71 (7.89 ± 1.50/calf)
Total cost of treatment (yen)	20,834 (2,604/calf)	26,281 (2,920/calf)

* Tendency of difference between the HK-LS HS-1 supplementation and control groups (p = 0.071).

** Tendency of difference between the HK-LS HS-1 supplementation and control groups (p = 0.093). HK-LS HS-1=Heat-killed *Lactobacillus sakei* HS-1, SE=Standard error

### Field trial to elucidate the effect of HK-LS HS-1 supplementation on F1 calf growth during the preweaning period (Experiment 2)

The mean body weights at calving in the supplemented (n = 98) and control (n = 130) groups were 42.9 ± 6.7 kg and 43.3 ± 6.6 kg, respectively, and no significant difference was observed between the two groups. During the experimental period, three calves (one in HK-LS HS-1 and two in the control group) died; thus, data from 225 calves were included in the present study. Table-2 shows the mean day interval between calving and weaning in both groups. The mean day interval between calving and weaning in female calves in the HK-LS HS-1 supplemental group (96.5 ± 11.4 days) tended to be shorter (p = 0.098) than that of the control calves (99.7 ± 11.2 days), and the total (male and female calves) day interval of the supplemental group (96.3 ± 10.6 days) tended to be shorter (p = 0.057) than that of the control group (98.7 ± 12.0 days).

**Table-2:** Effect of HK-LS HS-1 supplementation on weaning age of suckling F1 calves.

Groups	Male (mean ± SD)	Female (mean ± SD)	Total (mean ± SD)
Supplement (n = 97)	95.7 ± 10.5 (n = 62)	96.5 ± 11.4 (n = 35)*	96.3 ± 10.6 (n = 97)**
Control (n = 128)	98.1 ± 11.4 (n = 75)	99.7 ± 11.2 (n = 53)*	98.7 ± 12.0 (n = 128)**

* Tendency of difference between the HK-LS HS-1 supplementation and control groups (p = 0.098).

** Tendency of difference between the HK-LS HS-1 supplementation and control groups (p = 0.057). HK-LS HS-1=Heat-killed *Lactobacillus sakei* HS-1, SD=Standard deviation

## Discussion

In our previous report, we observed that supplementation with HK-LS HS-1 in the MR soon after birth reduced the incidence of disease during the 3-week feeding period and improved the intestinal microflora; thus, we speculated that HK-LS HS-1 has “prebiotic-like effects [16].” After weaning, when calf feed is changed from milk to solid feed, many diseases occur due to “weaning stress [3, 17, 19].” Therefore, we hypothesized that preliminary supplementation of HK-LS HS-1 1 month before the scheduled weaning period would improve the health condition of the calves to reduce the negative effects of weaning stress.

In Experiment 1, there was a trend toward ameliorating decreased appetite concomitant with reduced drug costs for treatments during the HK-LS HS-1 supplementation period from the clinical observations, but also substantial increases, such as that in T-Cho and Alb in the blood biochemical analysis, which may be metabolic parameters of nutritional status. Furthermore, based on the field results in Experiment 2, there was a substantial reduction in days from calving to weaning in the HK-LS HS-1-supplemented group compared to that in the control group, which may reflect the favorable growth and nutritional status of calves in the HK-LS HS-1 group compared to those in the control. Therefore, the results of this study may partially support our hypothesis regarding the positive effects of preliminary HK-LS HS-1 supplementation before weaning calves.

The beneficial effects of probiotics, which include preventing the growth of pathogenic bacteria, increasing digestive capacity, lowering the pH of the intestinal tract, and improving mucosal immunity, have been widely studied to improve the production and health of animals [9, 26]. In addition, because live *Lactobacillus* are difficult to preserve and their effect is not constant, attempts have been made to produce heat-killed *Lactobacillus*. The reported effects of orally administered, heat-killed LAB include the induction of IL-12, which leads to a T helper 1 type immune response; suppression of immunoglobulin E production against naturally-fed food allergies; and improvement in health-related quality oflife in mice [12, 15], humans [13, 14, 27, 28], and farm animals, such as pigs [27], and chickens [29, 30]. When HK-LS HS-1 supplementation was applied to farm animals, it improved the daily gain in body weight and the feed conversion of pigs [31]. The growth performance of broiler chickens was also improved, as HK-LS HS-1 supplementation improved the body weight gain and feed efficiency, which may be due to morphological changes in the hypertrophied intestinal absorptive epithelial cells on the villus apical surface [29]. We previously demonstrated the effects of HK-LS HS-1 supplementation in calves for the first time. We found a significant increase in LAB CFU/g at each successive sampling point, with fewer calves requiring medical treatment for diarrhea during the HK-LS HS-1 administration period [16]. Thus, although the HK-LS HS-1 supplementation period here differed from that of previous studies, the present results reproduce the beneficial effects of HK-LS HS-1 for the prevention of diseases in calves.

Although the cutoff value of SAA in calves during the milking period is unclear, we excluded one calf from the supplemented group due to a high SAA concentration (showing more than mean + 2 SD of the supplement group) of the day 0 sample to exclude suspiciously sick calves. The first experiment showed that daily HK-LS HS-1 administration for 30 days before weaning had etiotropic effects on the animal’s health, particularly appetite or diarrhea. It tended to reduce the number of calves with anorexia or diagnosed with and treated for diarrhea compared with the control group, although there was no substantial decrease in SAA between days 0 and 30 in the HK-LS HS-1 supplement group. These results may imply that enteric inflammation was mild in the supplemented group (reflected by the lack of significant changes in SAA results) compared with the control group receiving more medical treatments.

In Experiment 1, we used only female calves and focused on how weaning stress and HK-LS HS-1 supplementation affected AMH concentrations in the calves. The previous study on the AMH concentration of female JB cattle indicated a gradual increase with age after birth; typically, the SDs of the mean AMH concentration are larger at approximately 3 months of age than at any other age [22], which is assumed to be due to “weaning stress” in the approximately 3-month-old JB calves. We hypothesized that preweaning HK-LS HS-1 supplementation would reduce weaning stress and not adversely affect AMH levels before and after weaning. The results of this study discredited our hypothesis. Although no difference between days 0 and 30 in either group was observed, the AMH level in the control group was substantially increased after weaning and significantly increased on day 60 compared with that on day 0, while it tended to be stable in the HK-LS HS-1-supplemented group. We recently reported a negative correlation between AMH and SAA levels, demonstrating that inflammation is associated with decreased AMH levels in female cattle [32]. Therefore, the reason for differing trends in AMH levels between the two groups may be partially derived from differing preliminary trends of different inflammation statuses during the experimental period, which did not appear in the form of different SAA concentrations in the preweaning period between days 0 and 30. It may also partially be derived from the large SD range of AMH in the control group, compared to that in the supplement group, between days 0 and 30. Although, the AMH level on day 60 in the control group was higher than that in the control group on day 0 (p = 0.005) and lower than that in the supplement group, the SD value in the supplement group on day 60 (1 month after weaning) was 357.8, while that in the control group was 658.3, indicating that the SD value of the AMH concentration of the supplement group fluctuated less than that of the control group. Although it is difficult to examine the effect of HK-LS HS-1 supplementation or weaning itself against AMH concentration in the field without any clinical treatment, our results, especially for the control group that received more treatments than the supplement group as a real number during the HK-LS HS-1 supplemental period, indicate that this may be a suitable clinical treatment for sick calves and that it may prevent AMH reduction during the weaning period.

Hematological measurements were conducted to monitor the nutritional intake and health condition of HK-LS HS-1-supplemented calves compared to those of control calves. Although no differences were observed between the HK-LS HS-1 and control groups on any sampling day, the metabolic evaluation revealed substantial differences in some serum biochemical parameters within the HK-LS HS-1 group and those not within the normal reference ranges during the HK-LS HS-1 supplement period (between days 0 and 30), such as T-Cho and Alb, which are important parameters that reflect nutritional condition. In the present study, the GGT concentrations of both groups decreased substantially in each group in a time-dependent manner between days 0 and 30, as well as later on. As indicated by the previous studies by Perino *et al*. [33], Thompson and Pauli [34], and Wesselink *et al*. [35], the GGT concentration in newborn calves fed colostrum is usually extremely high (300 < U/L) and decreases in a time-dependent manner. Therefore, GGT concentrations may become stable (approximately 20 mg/dL) at the weaning age of JB calves (3 months).

In our previous report by Sasazaki *et al*. [16] regarding a 3-week supplementation of HK-LS HS-1 to calves soon after birth, the number of medications administered was lower (p < 0.05) in the supplement group (5.2 ± 3.9) than in the control group (10.6 ± 5.9) during the experimental period. Here, although the number of medications administered was similar between the two groups, the number of calves in the control group that left milk during the HK-LS HS-1 supplementation period tended to be higher than that in the supplemented group. The period from birth to weaning is stressful for calves, causing decreased immunity and reducing herd productivity [7, 10]. The intestines of newborn calves are sterile, and the colonization of the gastrointestinal tract begins immediately after birth [9]. Developing appropriate gastrointestinal tract microbiota in the early weeks of life is crucial for a functional immune system [36]. Therefore, considering the results of our previous report and this study, an important effect was observed compared to the control group; further, HK-LS HS-1 supplementation was also shown to be cost-effective. Supplementation with HK-LS HS-1 may be most effective when administered as early as possible after birth, when the intestinal flora is underdeveloped. The results of the field trial conducted in Experiment 2 also support this consideration, as the addition of HS-1 from the start of group rearing by a nursing robot, approximately 5 weeks after birth to the time of weaning, significantly reduced the age of weaning.

## Conclusion

The present study revealed the potential benefit of HK-LS HS-1 in improving the etiotropic effects and growth of calves during the preweaning period; however, it was also found that HK-LS HS-1 did not positively affect the AMH concentration compared with that in control calves after weaning. Future studies should be conducted to investigate the effects of continuous supplementation of HK-LS HS-1 after weaning to clarify the effect of HK-LS HS-1 on weaning stress reduction, such as health conditions, medical treatments, and cost-effectiveness.

## Authors’ Contributions

NS, TO, NI, DK, and MiT: Conceptualization. NS, US, OY, TO, TH, and MiT: Methodology. NS, KT, HH, DM, KI, and MiT: Sampling. OSW: Formal analysis. NS, OSW, MaT, and MiT: Writing-original draft. NS, OSW, MiT, OY, and TO: Writing-review and editing. All authors have read, reviewed, and approved the final manuscript.
